# A Design Methodology of Digital Control System for MEMS Gyroscope Based on Multi-Objective Parameter Optimization

**DOI:** 10.3390/mi11010075

**Published:** 2020-01-09

**Authors:** Haoyu Gu, Wei Su, Baolin Zhao, Hao Zhou, Xianxue Liu

**Affiliations:** Institute of Electronic Engineering, China Academy of Engineering Physics, Mianyang 621999, China; guhaoyu19901015@163.com (H.G.); suwei19901015@163.com (W.S.); chinamems@163.com (H.Z.); liuxx19901015@163.com (X.L.)

**Keywords:** MEMS gyroscope, closed-loop system, genetic algorithm, Monte Carlo analysis, Adam-LMSD algorithm

## Abstract

This paper presents a novel multi-objective parameter optimization method based on the genetic algorithm (GA) and adaptive moment estimation (Adam) algorithm for the design of a closed-loop control system for the sense mode of a Microelectromechanical systems (MEMS) gyroscope. The proposed method can improve the immunity of the control system to fabrication tolerances and external noise. The design procedure starts by deriving a parameterized model of the closed-loop of the sense mode. The loop parameters are then optimized by the GA. Finally, the ensemble of optimized loop parameters is tested by Monte Carlo analysis to obtain a robust optimal solution. Simultaneously, the Adam-least mean square (LMS) demodulator, which is appropriate for the demodulation of very noisy signals, is also presented. Compared with the traditional method, the time consumption of the design process is reduced significantly. The digital control system is implemented by the print circuit board based on embedded Field Programmable Gate Array (FPGA). The experimental results show that the optimized control loop has achieved a better performance, the system bandwidth in open-loop and optimal closed-loop control system is about 23 Hz and 101 Hz, respectively. Compared to a non-optimized closed-loop system, the bias instability reduced from 0.0015°/s to 7.52 × 10^−4^°/s, the scale factor increased from 17.7 mV/(°/s) to 23 mV/(°/s) and the non-linearity of the scale factor reduced from 0.008452% to 0.006156%.

## 1. Introduction

Microelectromechanical systems (MEMS) gyroscopes are widely used in various fields such as navigation, guidance and other measurement applications owing to low power consumption, low cost, and their small size [1]. However, due to the inherent fabrication tolerances which are caused by the imperfections of the fabrication process, the measurement accuracy and long-term bias instability of MEMS gyroscopes are degraded. In order to reduce the influence of fabrication imperfections, many techniques mainly focused on the the mechanical characteristics and fabrication process of the MEMS gyroscope have already been reported in previous studies [2,3,4,5,6,7,8,9]. In this paper, we focus on the design and optimization of the control system to improve the performance and adaptability of fabrication tolerances of the MEMS gyroscope. 

The traditional analog control systems suffer from external temperature drift and difficulties for implementing complex control strategies and demodulators [10]. In comparison, the digital control systems have the advantages of flexibility and insensitivity to variation of the environmental parameter and, therefore, the digital control systems have been widely used currently [11,12,13].

The gyroscope digital control system is mainly composed of the control loops and the demodulator. The control loops have two operation modes: open-loop operation and closed-loop operation. As previous studies showed, the digital closed control loops have considerable superiorities in terms of low-frequency noise immunity, linearity, bias stability and improved bandwidth [14,15,16]. The sigma-delta modulator loops, which are widely used in digital gyroscope closed control systems, can directly output digital stream signals that are easy to integrate in the post-digital process [17,18,19]. However, the stability of control loops may decrease due to the nonlinearity of the 1-bit quantizer as the amplitude of input signal increases [20,21]. Moreover, the sigma-delta loop becomes extremely complicated when the mechanical element of MEMS gyroscope is embedded in the closed loop. 

The control loop based on the Proportional-Integral (PI) controller is another effective method for a MEMS gyroscope [22,23]. Because of the demodulation and modulation processes, there are two closed loops in the system, which is called in-phase force rebalance control loop and quadrature suppression control loop. By contrast with the sigma-delta loop, there is no notch filter in control loops and the two loops are similar, which can not only simplify the realization of controllers but also improve the robustness of the control system [24,25]. However, the design of an optimized digital control loop for MEMS gyroscopes is still extremely complex as a multi-objective parameter optimization issue. In previously published works, the optimization method for MEMS gyroscope closed-loop control parameters is based on root locus techniques or directly on the transfer function [26,27]. Nevertheless, these approaches suffer from two disadvantages:

1. there still remains uncertainty regarding stability of the control loops, especially in the presence of variation and disturbance of practical parameters, which are relatively significant for MEMS sensors. Therefore, the typical design process has to be followed by massive system level simulation and parameter adjustment based on experimental results, which are time consuming;
2. the typical design is not optimized in terms of system performance, and there is an inability to evaluate how far the design differs from its optimum.

Therefore, a multi-objective optimization method based on the generic algorithm (GA) should be applied to the design and implementation of the gyroscope control loop based on PI controllers. The proposed method yields a design solution that is close to the optimum, as it takes into account integral of time and absolute error (ITAE) of the control loop which contains settling time and steady-state error as the index of performance evaluation. The robustness analysis performed following the GA is important, giving confidence in the design and ensuring manufacturability, and avoids the system becoming unstable because of inevitable fabrication tolerances. Monte Carlo analysis for a design is adopted to vary the structure parameters of gyroscope, and the mean value and standard deviation of ITAE in the designed control loop are calculated. A low mean value and standard deviation indicated a robust design. The design procedure starts by deriving the parameterized system-level model of a MEMS gyroscope closed loop based on the PI controller. The loop parameters are adaptive tuned by the GA within self-defined boundaries, and the performance of the control loop is evaluated.

As another important part of control system for a MEMS gyroscope, the demodulator also plays a crucial role in improving system performance [28]. The multiplication demodulators (MD) are widely used as a demodulation algorithm in a MEMS gyroscope digital control system [29]. However, multiplication demodulator has poor performance of noise immunity and harmonic suppression, which cannot be eliminated by a low-pass filter completely. In order to improve the noise performance of demodulator, Cheng and Li proposed a demodulator base on Fourier series [30]. Nevertheless, this demodulator is quite complex and spends much more logic resources. The least mean square demodulator (LMSD) is an adaptive algorithm which makes the demodulated signal approach a lower noise level and algorithmic complexity [31,32,33]. However, the choice of initial learning rate has a significant impact on the convergence time and stability, especially in the case of a noisy gyroscope detection signal. The adaptive moment estimation (Adam) algorithm, which was first presented in 2015 [34], is an effective method to improve the noise immunity in the process of algorithmic convergence. The initial learning rate can also be selected easily by the hyper-parameter optimization of Adam. In this paper, a novel, improved LMS demodulator based on the Adam algorithm (LMSD) is presented. Applying the algorithm to MEMS gyroscopes as a demodulator has not been mentioned in previous literature. The proposed demodulator is appropriate for the demodulation of weak signal from noise, and can achieve the lower noise level and high precision output of gyroscopes.

In order to achieve a better performance and adaptability of fabrication tolerances of the MEMS gyroscope, this paper proposes a design methodology for the digital control system combining the genetic-Monte Carlo algorithm and LMS demodulator with Adam optimization algorithm. To demonstrate the validity of the proposed design methodology, the optimized system level design of the PI-controller based closed-loop control system has been implemented as a Printed Circuit Board (PCB) circuit with embedded FPGA. Most of the control loop and demodulator is implemented in the FPGA platform.

This paper is organized as follows. Section 2 introduces the control loop design and optimization. Section 3 shows the basic principle and simulations of LMS demodulator with Adam algorithm, and several demodulator algorithms are also simulated to compare their performance. Section 4 gives the digital control system for the MEMS gyroscope. In order to verify the feasibility of the control loop designed in Section 2 and the Adam-LMS demodulator in Section 3, the circuit of the gyroscope control system is implemented and relevant experiments are carried out in Section 5. Finally, Section 6 concludes this paper.

## 2. The Design and Optimization of MEMS Gyroscope Control System 

### 2.1. The Linear Model of the Control System

**Figure 1:**
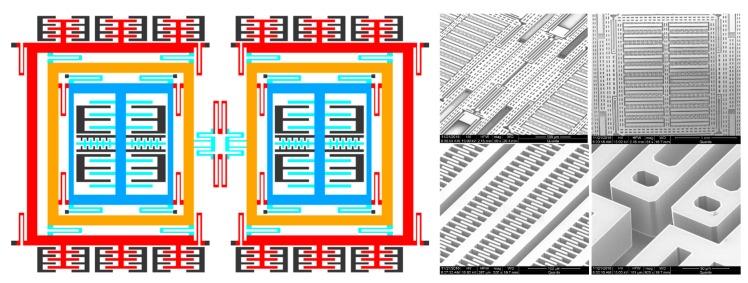
The schematic diagram of the tested Microelectromechanical systems (MEMS) gyroscope and a scanning electron micrograph (SEM) of the sensing element of the gyroscope.

The z-axis tuning fork MEMS gyroscope in our laboratory is presented in [35], which is fabricated in a Silicon on Insulator (SOI) process, as shown in Figure 1. The total dimension of the sensing element 4 mm × 8 mm.

The driving voltage, which consists of the alternating current (AC) voltage and direct current (DC) bias, is applied to the driving electrodes excite the proof mass to vibrate along the x-axis at drive mode resonant frequency; According to the principle of the Coriolis effect, the Coriolis force is generated by an input angular rate around the z-axis, and the proof mass is driven to vibrate along the y-axis. The vibratory displacement of proof mass results in a capacitive signal, detected by the sensing electrodes, which is processed by the interface circuit and demodulator to obtain the input angular rate. The drive mode contains two groups of combs, which are called drive combs and drive-sensing combs. Similarly, two groups of combs are applied to the sense mode, which are called sense combs and force feedback combs. The drive-sensing combs and the feedback combs are applied to closed-loop control of drive and sense mode respectively. In this paper, we focus on the control loop of sense mode. 

**Figure 2:**
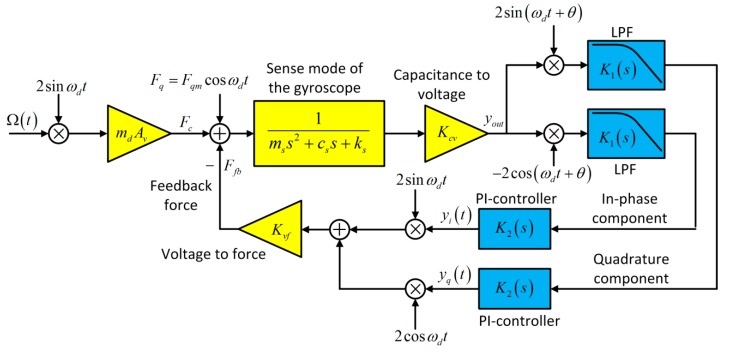
The schematic diagrams of force feedback control loop for sense mode of the MEMS gyroscope.

The force feedback control loop for sense mode based on PI-controllers is shown in Figure 2. Given the demodulation and re-modulation process, there are two rebalance loops in the control loop, which are called in-phase rebalance loop and quadrature suppression loop. To ensure the completeness of the control system, demodulation and re-modulation signals in the two loops should be orthogonal. Through the PI-controllers, the re-modulation process should be adopted to construct the feedback force to rebalance both Coriolis force and quadrature component generated by the drive mode coupling. As the final output signal of the closed control loop of the sense mode, the in-phase component $y_{i}\left(t\right)$ contains the information of input angular rate. Different from the sigma-delta loop, there is no notch filter in control loops and the two loops are similar, which can not only simplify the realization of controllers but also improve the robustness of the control system. The basic analytical model of the closed control loop should be derived to provide crucial guidance for the design and optimization.

Assume that the input angular rate $\Omega_{z}$ is equal to $\Omega_{R}cos(\omega_{R}t)$ and the driving velocity $v_{d}$ is $A_{v}sin(\omega_{d}t)$; where $\omega_{d}$ represents the natural frequency of the drive axis, and the amplitude of drive velocity $A_{v}$ is constant, which can be guaranteed by the control loop of drive mode. The dynamic model of the MEMS gyroscope is equivalent to a “mass-spring-damper” second-order mechanical vibration system. As to the decoupled symmetric MEMS gyroscope, the dynamic equation of sense mode is shown as follows:

(1) $$m_{s}\ddot{y}+c_{y}\dot{y}+k_{y}y=-2m_{d}\Omega_{z}v_{d}$$

where m represents the sense mass of the gyroscope. $c_{y}$ and $k_{y}$ are the damping constant and spring stiffness constant of the sense mode, respectively. y is the displacement in the sense direction. Then, the Coriolis force is described as:

(2) $$F_{c}=-2m_{d}\Omega_{z}v_{d}=-F_{cm}cos\left(\omega_{R}t\right)sin\left(\omega_{d}t\right)$$

where $F_{cm}=-2m_{d}A_{v}\Omega_{R}$ represents the amplitude of Coriolis force. 

Then, the output voltage signal $y_{out}$, which is generated by the displacement along the sense axis, can be written as:

(3) $$\begin{array}{c} y_{out}=\frac{F_{cm}}{2m}[A_{1}sin\left(\omega_{d}+\omega_{R}\right)t+B_{1}cos\left(\omega_{d}+\omega_{R}\right)t+A_{2}sin\left(\omega_{d}-\omega_{R}\right)t\\ +B_{2}cos\left(\omega_{d}-\omega_{R}\right)t] \end{array}$$

The $\omega_{R}$ is a public variable of $A_{1}$, $A_{2}$, $B_{1}$ and $B_{2}$, as described in Equations (4)–(7):

(4) $$A_{1}=\frac{\omega_{s}^{2}-{\left(\omega_{d}+\omega_{R}\right)}^{2}}{{\left[\omega_{s}^{2}-{\left(\omega_{d}+\omega_{R}\right)}^{2}\right]}^{2}+\omega_{s}^{2}{\left(\omega_{d}+\omega_{R}\right)}^{2}/Q_{s}^{2}}$$

(5) $$B_{1}=\frac{-\omega_{s}\left(\omega_{d}+\omega_{R}\right)/Q_{s}}{{\left[\omega_{s}^{2}-{\left(\omega_{d}+\omega_{R}\right)}^{2}\right]}^{2}+\omega_{s}^{2}{\left(\omega_{d}+\omega_{R}\right)}^{2}/Q_{s}^{2}}$$

(6) $$A_{2}=\frac{\omega_{s}^{2}-{\left(\omega_{d}-\omega_{R}\right)}^{2}}{{\left[\omega_{s}^{2}-{\left(\omega_{d}-\omega_{R}\right)}^{2}\right]}^{2}+\omega_{s}^{2}{\left(\omega_{d}-\omega_{R}\right)}^{2}/Q_{s}^{2}}$$

(7) $$B_{2}=\frac{-\omega_{s}\left(\omega_{d}-\omega_{R}\right)/Q_{s}}{{\left[\omega_{s}^{2}-{\left(\omega_{d}-\omega_{R}\right)}^{2}\right]}^{2}+\omega_{s}^{2}{\left(\omega_{d}-\omega_{R}\right)}^{2}/Q_{s}^{2}}$$

where $\omega_{s}$ and $Q_{s}$ are the natural frequency and quality factor of the sense axis, respectively. $K_{cv}$ represents the conversion gain from capacitance to voltage, and $K_{vf}$ is the conversion gain of voltage to force. The output voltage signal $y_{out}$ is demodulated by the reference signal of $-2cos\left(\omega_{d}t+\theta \right)$, which is generated from the drive’s closed control loop. Then the demodulated signal passes through the low-pass filter to eliminate the high frequency components, which is shown in Figure 2. The in-phase component is written as follows:

(8) $$y_{in}=-\Omega_{R}\left(Asin\omega_{R}t+Bcos\omega_{R}t\right)$$

where

(9) $$A=A_{v}K_{cv}\left(A_{1}cos\theta -A_{2}cos\theta +B_{1}sin\theta -B_{2}sin\theta \right)$$

(10) $$B=A_{v}K_{cv}\left(B_{1}cos\theta +B_{2}cos\theta -A_{1}sin\theta -A_{2}sin\theta \right)$$

Therefore, the transfer function of the in-phase path can be obtained as Equation (11) by using the Laplace transforms on Equation (8):

(11) $$SF_{open}\left(s\right)=-B+Aj=-B+As/\omega_{R}$$

where $s=j\omega_{R}$.

Considering the transfer function of low-pass filter, the linear model of the open loop transfer function G(s) can be obtained:

(12) $$G\left(s\right)=SF_{open}\left(s\right)\times K_{1}\left(s\right)=-B+Aj\times \frac{\omega_{0}^{2}}{s^{2}+2\xi_{0}\omega_{0}+\omega_{0}^{2}}$$

where $\omega_{0}$ and $\xi_{0}$ are the cut-off frequency and damping coefficient of the second-order LPF $K_{1}\left(s\right)$, respectively. The simplified PI controllers are adopted in both quadrature suppression loop and in-phase rebalance loop, and the two PI controllers are identical. The simplification of the identical PI controller can reduce the complexity of implementation and improve the robustness of the control system further. The transfer function of the selected PI controller can be given as below:

(13) $$K_{2}\left(s\right)=\frac{k\left(s+\omega_{2}\right)}{\left(s+\omega_{1}\right)\left(s+\omega_{3}\right)}$$

The portion $\left(s+\omega_{2}\right)/\left(s+\omega_{1}\right)$ can be viewed as a PI controller, and the portion $k/\left(s+\omega_{3}\right)$ is a first order LPF applied to filter the high-frequency components. Then, according to Figure 1, the transfer function of the open loop of the sense mode is shown as follows:

(14) $$L\left(s\right)=y_{i}\left(s\right)/\Omega \left(s\right)=G\left(s\right)\times K_{2}\left(s\right)$$

Finally, the transfer function of the closed loop of the sense mode can be obtained in Equation (15):

(15) T(s)=L(s)/(1+L(s))

The linear model of the control loop, which is based on the transfers function, is an important foundation and prerequisite for the optimization and robustness analysis in the following. 

### 2.2. The Genetic Algorithm (GA)

**Figure 3:**
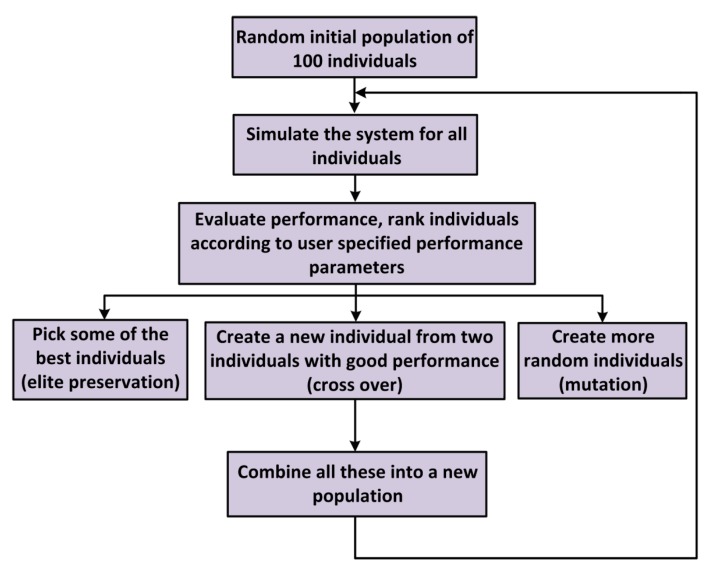
The flow chart of the genetic algorithm (GA) applied to optimize the control loop parameters.

Genetic algorithms (GA) are evolution algorithms inspired by the natural selection mechanics in the biology, and were presented by Holland in 1975 firstly [36] to solve multi-objective optimization problems. Genetic algorithms are appropriate for particularly complex multi-objective optimization problems, and it is capable of finding optimal solutions in a short period of time. The flow chart of the GA adopted in this work is shown in Figure 3 [37].

The parameters of the linear transfer function that are to be optimized (highlighted in blue in Figure 1) compose the set of parameters, namely an individual. In this paper, it takes into account integral of time and absolute error (ITAE) of the control loop which contains settling time and steady-state error as the index of performance evaluation. For each individual in the population, a MATLAB simulation based on the linear transfer function model of the control loop is carried out. The process of the genetic algorithm continues until the specified maximum number of generations is reached, and the final solution is the fittest and most evolved. In this paper, the algorithm generates 100 individuals in one population and simulation length is 40 generations. The key parameters of the MEMS gyroscope manufactured in our laboratory are shown in Table 1.

**Figure 4:**
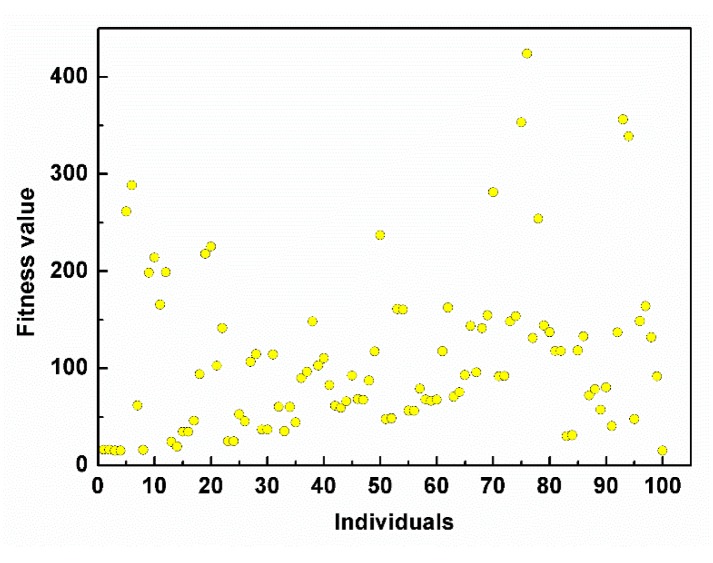
The scatter plot of the integral of time and absolute error (ITAE) performance for all the generated individuals.

The scatter plot of the ITAE performance for all the generated individuals is shown in Figure 4.

**Figure 5:**
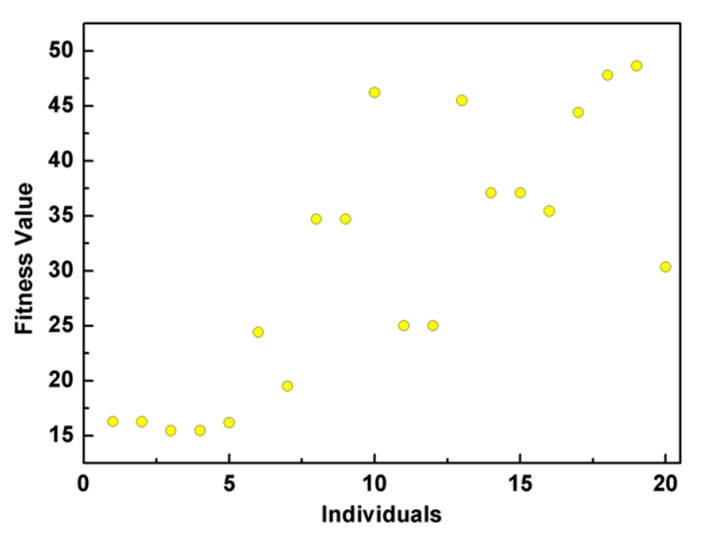
The scatter plot of the first 20 individuals with lowest ITAE.

After the process, the first 20 individuals with the lowest ITAE values were selected, as shown in Figure 5, and the robustness analysis was performed to choose one individual for hardware implementation.

### 2.3. The Robustness Analysis Based on Monte Carlo Algorithm

**Figure 6:**
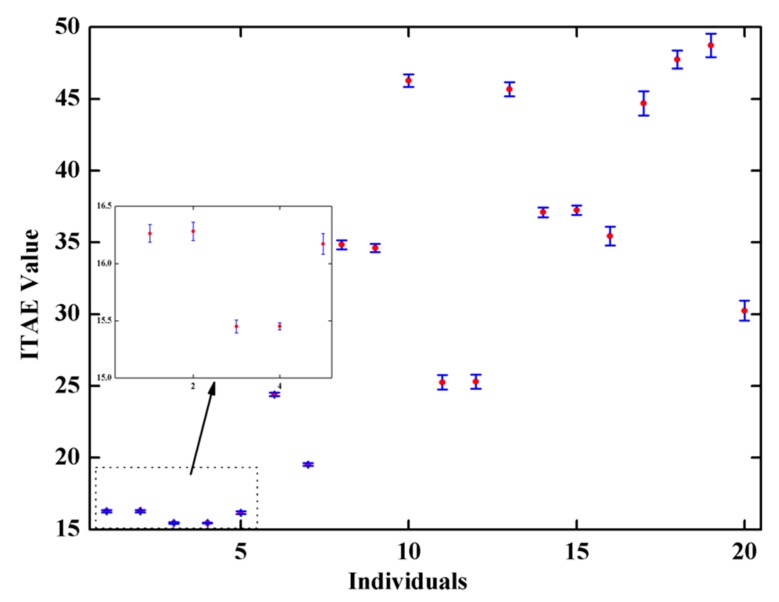
The statistical error bar of the mean of resulting ITAE with standard deviation.

Due to the limitation of the fabrication process technology, MEMS gyroscope is inevitably subjected to relatively large fabrication tolerances. Small variations of mechanical structure geometry cause fluctuations of the lumped parameters of the gyroscope in the range of ±20% their nominal values in general. The ITAE performance of some individuals, despite satisfying the requirement of objective function criteria, is especially susceptible to parameter fluctuations. Therefore, it is crucial to perform a fluctuation analysis and guarantee the final result is robust. Such an analysis is generally performed by Monte Carlo simulations. A specified standard deviation is given to each parameter of the individuals, and an ensemble of these parameters which are statistically fluctuated around their nominal value. Then, a specified number of Monte Carlo simulations are carried out and the results are plotted as two statistical histograms of the mean and the standard deviation of the resulting ITAE, respectively, as shown in Figure 6.

Here, the number of Monte Carlo simulations is set as 100 for each of the 20 selected individuals, and the parameters of the sensing element are fluctuated by 20% to reflect the significant fabrication tolerances that a MEMS gyroscope usually performs. From Figure 5, the loop parameters in the 4th individuals are chosen as optimal solution, and the mean value of ITAE in the closed control loop for sense mode was 15.45 with a standard deviation of 0.03. The final optimal parameters are selected to be taken forward for hardware implementation in Section 4. 

## 3. Least Mean Square (LMS) Demodulator with Adam Optimization Algorithm

**Figure 7:**
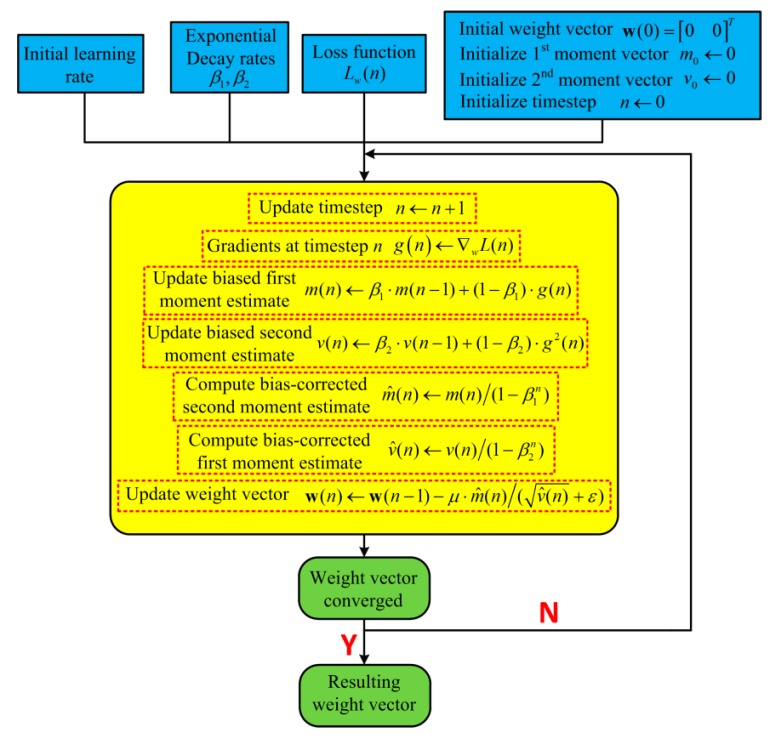
The flowchart of the least mean square (LMS) demodulator based on adaptive moment estimation (Adam) optimization algorithm.

To improve noise performance and the robustness of inevitable fabrication tolerances of the control system further, The Adam-LMS demodulation algorithm which is appropriate for the demodulation of very noisy signal is adopted. The flow chart of the algorithm is shown in Figure 7. 

The algorithm updates the exponential moving average of gradient m(n) and gradient square v(n) of loss function, which are obtained by using biased first and second moment estimation respectively. The exponential decay rates of m(n) and v(n) are adjusted by hyper-parameter $\beta_{1}$ and $\beta_{2}$ respectively. The initial value of m(n) and v(n) are zero, which will cause moment estimates to approach zero, especially in the case of small initial time-step and decay rate. Therefore, the initial value of m(n) and v(n) should be corrected and the bias-corrected moment estimate can be computed as shown in Figure 6. Utilizing the exponential moving average method, the effect of noise on gradients in the convergence process of weight vectors can be suppressed. In addition, $\hat{m}\left(n\right)/\sqrt{\hat{v}\left(n\right)}$ in the update step size can be defined as the signal-to-noise ratio (SNR) of the convergence process, hence the step size is adaptive to SNR, which can avoid the divergence of weight vectors due to the influence of noise.

**Figure 8:**
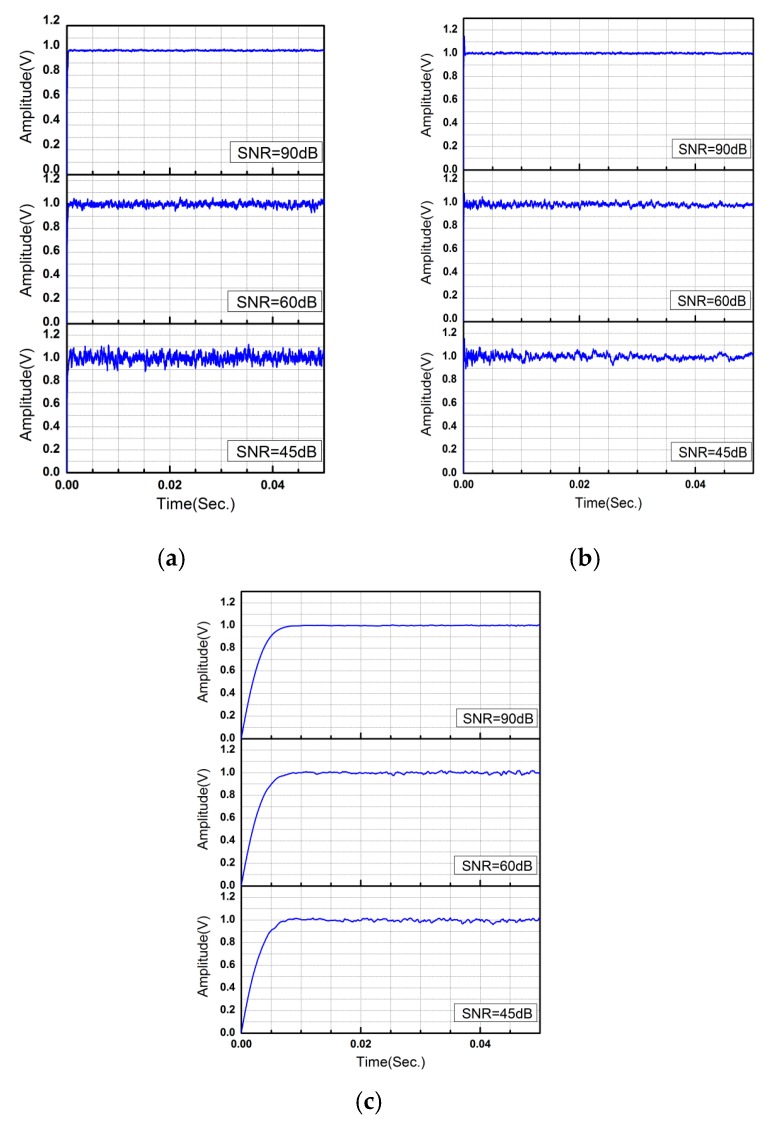
The demodulation results of (**a**) multiplication demodulator; (**b**) LMS demodulator; (**c**) Adam-LMS demodulator under different signal-to-noise ratio (SNR) conditions.

The three demodulation algorithms, including MD, LMSD and Adam-LMSD, are simulated by MATLAB to compare their performances. The input signal s(t) is a sinusoidal wave with the amplitude of 1 V and the frequency of 8 kHz, which is superposed with a Gaussian white noise. The results of different demodulators under different SNR conditions are shown in Figure 8. 

As shown in Figure 8a,b respectively, the phase and amplitude of the detection signal can be demodulated and obtained by using the multiplication demodulator and the LMS demodulator at a relative high SNR level of detection signal. However, when the SNR of the detection signal becomes lower, the multiplication and LMS demodulator are both difficult to converge, and the information of phase and amplitude obtained by demodulation contains a large volume of noise, which affects the stability and measurement accuracy of the gyroscope control system heavily. From Figure 8c, we can obtain that the Adam-LMSD has a better performance in the demodulation of phase and amplitude under a low SNR level, but a slightly longer convergence time. 

**Figure 9:**
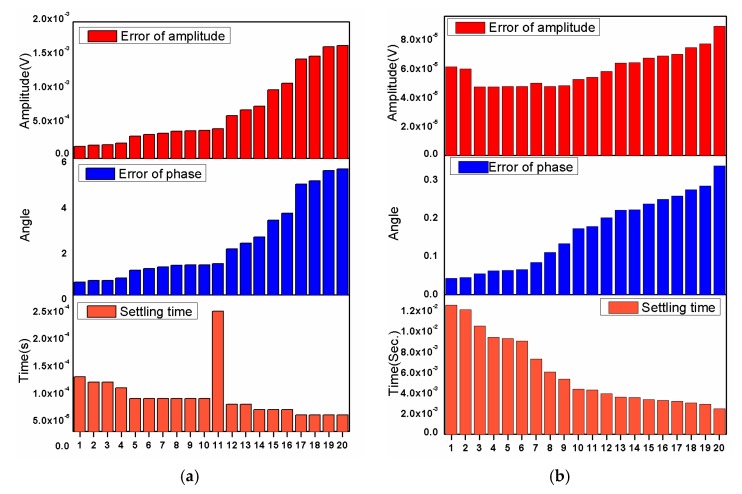
The demodulation amplitude error, phase error and convergence time of (**a**) LMS demodulator; (**b**) Adam-LMS demodulator under different initial learning rates.

To verify the robustness of the demodulator to the initial learning rate, the two demodulators, including LMS demodulator and Adam-LMS demodulator, are simulated by MATLAB on a personal computer. The input signal is still selected as s(t), and the SNR of the input signal is set as 45 dB. Within the interval from 0.01 to 0.1, 40 different values are generated randomly as the initial learning rate of the two demodulators, and the demodulation error of phase and amplitude and convergence time are taken as three indexes to evaluate the performance of the demodulator. The simulation results are shown in Figure 9a,b, respectively. It can be concluded from Figure 9 that the Adam-LMS demodulator has better robustness to the initial learning rate.

## 4. The Control System Implementation

**Figure 10:**
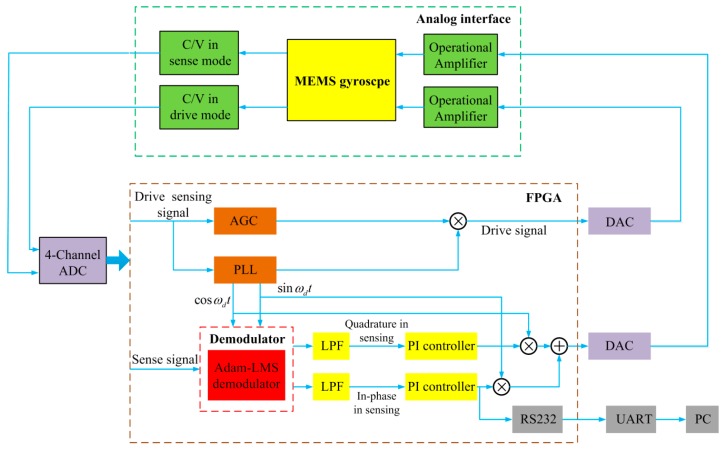
The digital closed-loop control system of the MEMS gyroscope.

The optimized closed control loop based on the PI-controller and the Adam-LMS demodulator are described in this paper, and the MEMS gyroscope digital control system is shown in Figure 10.

The system consists of analog interface circuit, Adam-LMS demodulator and the digital control circuit based on the PI-controller. As mentioned above, the two key parts of the gyroscope control system are the digital control circuit and Adam-LMS demodulator. The analog interface circuit contains capacitance/voltage conversion circuit and front-end amplifier. The output signals of drive mode and sense mode are controlled by two orthogonal re-modulation signals generated by the FPGA separately. The detected signals of both drive and sense modes are converted into the FPGA by using a 4-Channel analog-to-digital converter (ADC) to obtain the amplitude and phase information. In the drive mode, the amplitude and phase of the drive detection signal are applied to guarantee the drive closed control loop stably. To ensure the MEMS gyroscope is vibrating under constant amplitude and fixed phase conditions, the proposed control system adopts automatic gain control method to track amplitude and phase-locked loop method to stabilize phase and frequency. In the control loop of the sense mode, the Adam-LMS demodulator can extract the in-phase Coriolis signal and quadrature signal respectively. The control loop of the sense mode utilizes the force rebalance closed-loop detection technology. Through the PI-controllers, the re-modulation process is adopted to construct the feedback force to rebalance both Coriolis force and quadrature component generated by the drive mode coupling. As the final output signal of the closed control loop of sense mode, the angular rate output signal is shown on the computer through RS232.

## 5. Experiment Verification

**Figure 11:**
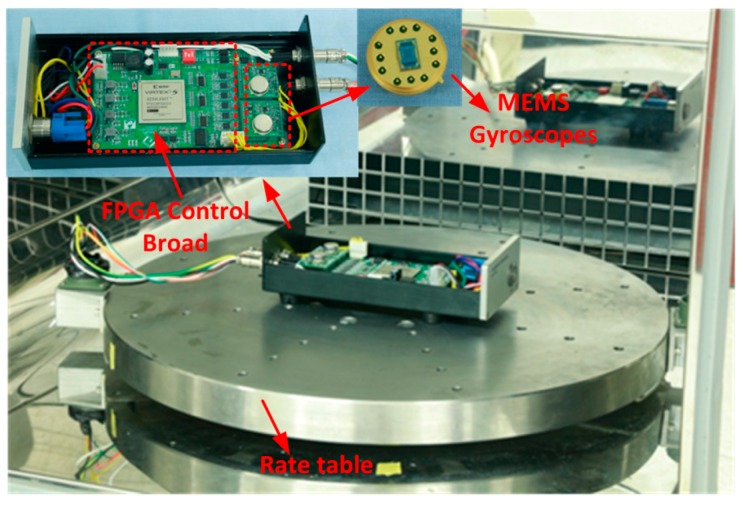
The PCB (Printed Circuit Board) circuit embedded with FPGA (Field Programmable Gate Array) for the MEMS gyroscope control system and experimental equipment.

In order to verify the feasibility and performance of the proposed design methodology based on multi-objective parameter optimization for the MEMS gyroscope closed-loop control system, the PCB circuit embedded with the FPGA of the control system is implemented as described in Section 4, and the relevant experiments are carried out. Figure 11 shows the gyroscope control circuit and experimental equipment.

The tested MEMS gyroscope manufactured in our laboratory has been described in Section 2. In the specific electronics, a 4-channel 24-bit resolution ADC ADS131 (Texas Instruments Company, Dallas, TX, USA) and two 16-bit DACs AD5724 (Analog Device, Norwood, MA, USA) are adopted to ensure lower quantization of noise and higher sampling rate for both sense and drive mode. A high-performance FPGA chip XC5VLX (Xilinx Company, San Jose, CA, USA) is used to satisfy the requirements of the logic elements and reduce the power consumption. The angular rate output can be transmitted to a personal computer through RS232.

**Figure 12:**
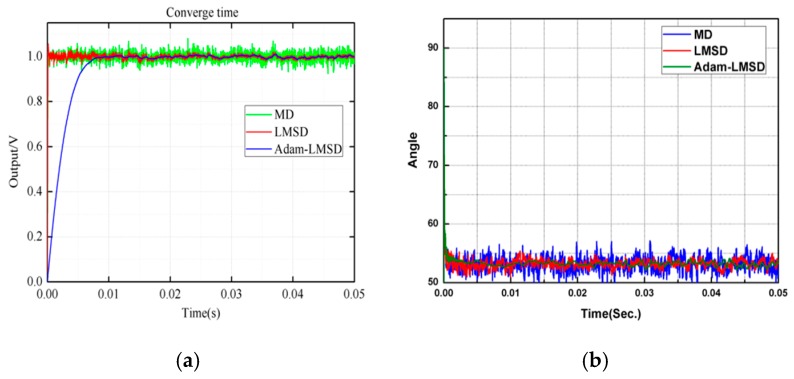
The comparison of (**a**) amplitude and (**b**) phase information of the demodulation results obtained by three different demodulators.

The experimental verification of the three demodulation algorithms is carried out and the reference angular rate signal is generated by a high precision rate table. The demodulation results of amplitude and phase are shown in Figure 12a,b respectively. We can find that the Adam-LMSD has lowest noise level and a better ability to attenuate the harmonics.

**Figure 13:**
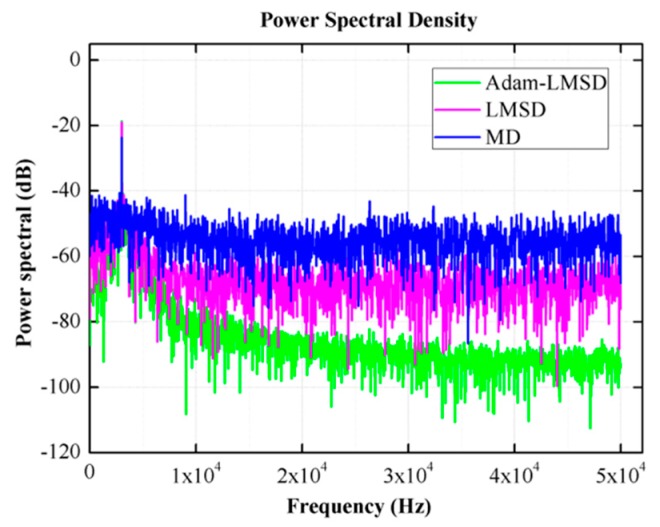
The diagram of power spectral density of three demodulators.

Figure 13 shows the power spectral density of three demodulation algorithms. We can find that the Adam-LMSD has lowest noise level and a better ability to attenuate the harmonics.

**Figure 14:**
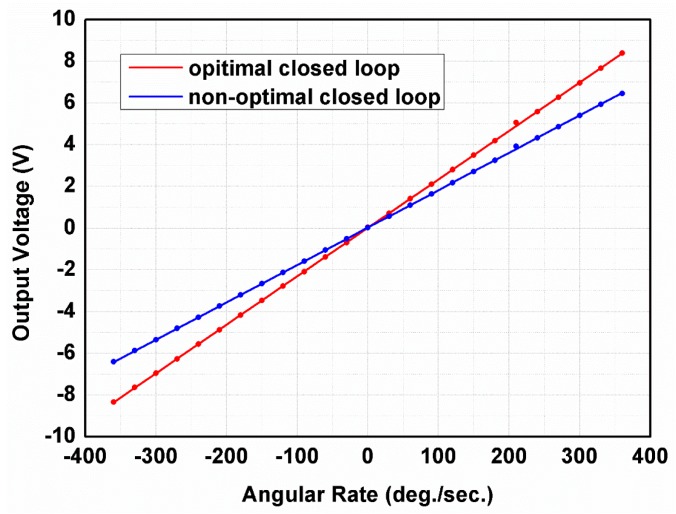
The measured results of gyroscope’s output for different reference angular input rate signals with a range of ± 360°/s in non-optimal and optimal closed-loop control system.

As a comparative example, the non-optimal closed-loop control system was implemented by randomly varying the loop parameters value, with a range of 10% of the optimized value. The high precision rate table was applied to generate the reference angular rate signal for measurement of the scale factor and the non-linearity. The measured results of the gyroscope’s output for different reference angular input rate signals with a range of ±360°/s in non-optimal and optimal closed-loop control system are illustrated in Figure 14. 

**Figure 15:**
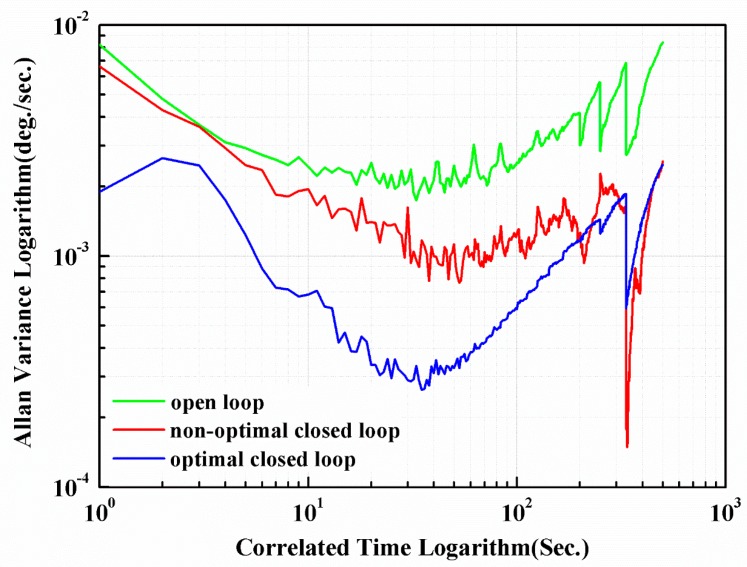
The Allan variance curves of the tested gyroscope in three different control systems.

The non-optimal closed-loop control system is still stable, but its performance is worse. The measured scale factors of non-optimal and optimized closed-loop system are 17.7 mV/(°/s) and 23 mV/(°/s), respectively. The non-linearity of the tested MEMS gyroscope in non-optimal and optimal closed-loop control system is 0.008452% and 0.006156%, respectively. The Allan variance curves [38] of the gyroscope in open, non-optimal and optimized closed-loop control systems are shown in Figure 15.

Utilizing quantitative analysis of Allan variance, we can determine that the bias stability are 0.0049°/s, 0.0015°/s and 7.52 × 10^−4^°/s, and the angle random walk are 1.047 × 10^−4^°/$\sqrt{s}$, 7.984 × 10^−5^°/$\sqrt{s}$ and 6.47 × 10^−5^°/$\sqrt{s}$, for 1000 s measurements of open-loop, non-optimal and optimal closed-loop systems, respectively. 

**Figure 16:**
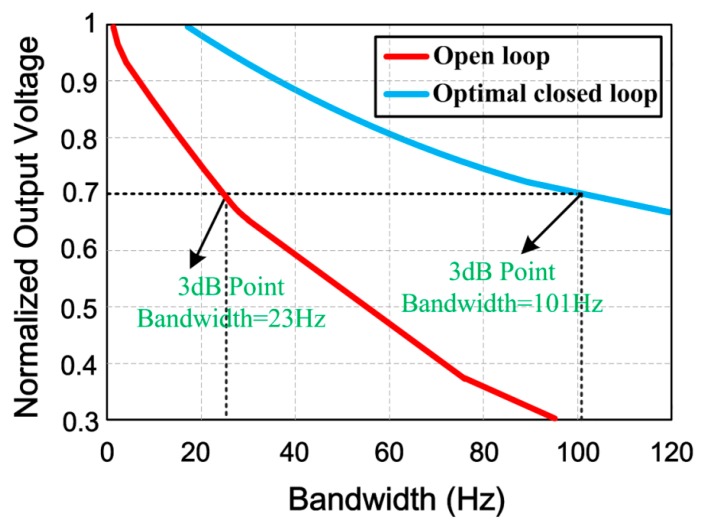
Frequency response of the open-loop and optimized closed-loop control systems.

Figure 16 illustrates the frequency response of the open-loop and optimized closed-loop control systems. The experimental results show that the system bandwidth in open-loop and optimal closed-loop control systems is about 23 Hz and 101 Hz, respectively.

The overall measured performance of the optimal gyroscope system is compared with non-optimal system and with other gyroscope control systems reported previously [10,35], and the results are summarized in Table 2. It should be noted that the application of the proposed optimal closed-loop system enhances the performance significantly.

## 6. Conclusions

This paper proposes a multi-objective parameter optimization method based on a genetic algorithm for the design of the closed control loop and a LMS demodulator with Adam optimization algorithm, and the robustness of the optimized parameters has been analyzed by the Monte Carlo method.

The control loop and demodulator are the two most important parts of the MEMS gyroscope control system. By using the genetic algorithm, the optimized solutions within a specified parameter range can be obtained; here, the integral of time and absolute error (ITAE) of the control loop which contains settling time and steady-state error are selected as the index of performance evaluation. The robustness of optimized parameter ensembles was analyzed by the Monte Carlo algorithm in order to choose the solution that is both optimal and robust. The proposed design methodology reduces the time consumption and complexity of the design process, and guarantees the performance of the control loop even when fabrication tolerances existed. 

As another important part of the control system for MEMS gyroscope, the LMS demodulator based on Adam optimization algorithm is proposed in this paper. The Adam-LMS demodulator is the effective method to demodulate weak signals from heavy noise, having strong noise suppression properties. MATLAB simulations have been performed to verify the performance of the Adam-LMS demodulator, which is the same as the theoretical analysis. Compared with other demodulator, the Adam-LMS demodulator has the advantages of a lower noise level and the better performance in harmonics attenuation.

Combining the optimized design of the closed control loop and the Adam-LMS demodulator, the closed-loop control system of the sense mode of the MEMS gyroscope fabricated in our laboratory has been implemented in a hardware circuit embedded with FPGA. The experimental results show that the bias instability of the tested MEMS gyroscope achieved 7.52 × 10^−4^°/s and the non-linearity of the scale factor reduced from 0.008452% to 0.006156%. Compared with the open-loop operation, the bandwidth of the gyroscope closed-loop control system increased from 23 Hz to 101 Hz.

Consequently, the optimized closed-loop control system with Adam-LMS demodulator for a MEMS gyroscope is feasible and effective.

## Figures and Tables

**Table 1:** The key parameters of the tested gyroscope.

Parameter	Values
Drive mode resonant frequency	8085 Hz
Sense mode resonant frequency	8042 Hz
Quality factor of drive mode	384
Quality factor of sense mode	395
Drive effective mass	6.39 × 10^−7^ kg
Sense effective mass	6.2 × 10^−7^ kg
Capacitance of drive mode	9 pF
Capacitance of sense mode	8 pF

**Table 2:** The comparison results between the proposed method and conventional methods.

	Non-Optimal Closed Loop	Optimal Closed Loop	Ref. [35]	Ref. [10]
$Q_{x}$	384	384	386	5500
$Q_{y}$	395	395	345	7000
Bias stability (°/s)	0.0015	7.52 × 10^−4^	0.007	0.0034
Scale Factor (mV/(°/s))	17.7	23	6.9	9.84
Non-linearity (%)	0.008452	0.006156	0.09	0.06
Angle random walk $\left({}^{\circ}/\sqrt{s}\right)$	7.984 × 10^−5^	6.47 × 10^−5^	2.6 × 10^−4^	1.8 × 10^−4^
